# A Comparative Study of Biocompatibility in Rat Connective Tissue of a New Mineral Trioxide Compound (Theracal) versus MTA and a Bioactive G3 Glass

**DOI:** 10.3390/jcm10122536

**Published:** 2021-06-08

**Authors:** Jesús Mena-Álvarez, Cristina Rico-Romano, Carlos Gutiérrez-Ortega, Pablo Arias-Sanz, Javier Castro-Urda

**Affiliations:** 1 Department of Endodontics, Faculty of Dentistry, Alfonso X El Sabio University, 28691 Madrid, Spain; cromaric@uax.es; 2 Department of Epidemiology, Central Defense Hospital “Gomez Ulla”, 28047 Madrid, Spain; cgutort@oc.mde.es; 3 Veterinary Service, Central Defense Hospital “Gomez Ulla”, 28047 Madrid, Spain; parisan@oc.mde.es; 4 Department of Veterinary, Faculty of Veterinary, Alfonso X El Sabio University, 28691 Madrid, Spain; j.castro.u@gmail.com

**Keywords:** mineral trioxide, G3 glass bioactive, Theracal, root-end sealers, endodontic surgery

## Abstract

The aim of this paper was to assess the differences in tissue response to implantation during 15, 30 and 45 days in the subcutaneous connective tissue of Wistar rats from three biomaterials: Angelus MTA^®^, Theracal LC^®^, and Angelus MTA^®^ to which 25% bioglass G3 was added. Twenty-four Wistar rats were used, the materials were inserted into the rat’s dorsal area in silicone tubes 5 mm long by 1.5 mm diameter. Histological reaction was assessed at 15, 30, and 45 days after implantation. They were then stained with hematoxylin eosin and evaluated by two observers. Data were analyzed using Fisher’s exact test and Mann–Whitney’s U test was used to determine the association between variables. Angelus MTA induced the formation of dystrophic calcifications twice as much as Theracal LC (*p* < 0.05). The addition of G3 did not affect the greater or lesser occurrence of calcifications (*p* > 0.05). Theracal LC and MTA plus G3 caused an inflammatory reaction, which was chronic at 15 days and decreased in intensity, almost disappearing after 45 days. Theracal LC, as well as Angelus MTA plus G3, were well tolerated when implanted in the subcutaneous connective tissue of rat. Histologically, no inconvenience was found for the use by direct contact of Theracal LC, and the mixture of MTA with 25% bioactive glass G3, in the tissue of Wistar rats.

## 1. Introduction

Endodontic microsurgery is often the last option when non-surgical retreatment fails, is unfeasible or unlikely to improve the initial endodontic treatment. In particular, only surgical intervention may resolve cases involving a persistent lesion (microbial infection) with etiology related to complex canal anatomy for this reason.

Root-end filling materials in endodontic microsurgery have evolved over time. However, these materials have the drawback of suffering from corrosion, electrolysis, expansion and coloration. They also present filtration problems, sealing deficiencies and marginal adaptation, show difficulties in their handling, the alteration of their setting in the presence of humidity and toxicity to vital tissues [1].

Microorganisms play an essential role in periapical disease [2], so the antibacterial properties of these materials are essential when assessing their suitability. The aim of these materials is to achieve an airtight seal after the resection of the apical root zone [3], sealing the communication between the periapical tissue and the root canal of the tooth. The root-end filling materials will be in direct contact with the periapical tissue, so they must be biocompatible [3] in order to prevent any response that conditions the failure of endodontic therapy [4]. The four classic methods that have been used to study the biocompatibility of materials have been: the evaluation of biocompatibility, placement of subcutaneous implants, placement of intraosseous implants, and in vivo evaluation of the periradicular tissue reaction in human subjects [5].

Mineral trioxide aggregate (MTA) has been used as a root-end filling material since the beginning of its presentation and its biocompatibility has been extensively analyzed [4,6,7,8,9,10].

Theracal LC^®^ (Bisco Inc, Schamburg, IL, USA) is a material used in pulp coatings. It is a light-curing, resin-modified calcium silicate cavity liner designed to act as a barrier [11]. Theracal LC^®^ is composed of 45% Portland cement type III, 10% radiopaque material, 5% fumed silica (hydrophilic thickening agent), and 40% resin (it contains different hydrophobic monomers and hydrophilic monomers) [12]. Nor is there bibliography that analyzes the biological properties of Theracal LC^®^ as a root-end filling material, although the rest of its properties have been analyzed [12,13,14,15,16,17,18,19]. This material is light-curing and improves the two main disadvantages of MTA, which are the setting time and the difficulty in handling.

Bioactive glasses have evolved to the fourth generation or P2O5-free bioglasses. The improvements of the fourth-generation bioglasses open the door to the development of new applications in the dental field, such as periodontics, surgery, implantology, prosthetic rehabilitation and in endodontic treatments. Among the bioglasses is G3; it is a sodium–calcium glass (soda–lime) similar to Hench’s glass, but is free of phosphate, with a composition SiO2-Na2O-Al2O3-CaO-B2O3. López-Píriz et al. [20] used G3 to coat implants that they placed in Beagle dogs, observing improved periodontal health. The authors state that G3 bioglass is capable of preventing bone resorption around the implanted material. Studies with G3 impellers implanted in the jaws of Beagle breed dogs show that the rate of resorption of these fourth-generation glasses, for a porosity of 17% of the total weight, is adapted to the rate of bone growth. Not only is there bone growth in the resorption space of the G3 bioglass, but the histological sections demonstrate a cellular invasion within the porosity of the rim [21]. If we added it to other materials, the biocidal capacity of MTA could be strengthened, and its chemical and mechanical properties would be improved.

The aim of this study was to compare the biocompatibility of MTA Angelus^®^ (Angelus, Londrina, Brazil), Theracal LC^®^, and MTA Angelus^®^ with G3 bioactive glass particles in a known ratio when the materials were implanted into the subcutaneous connective tissue of Wistar rats.

## 2. Materials and Methods

### 2.1. Study Design

The present research project has been approved by the Ethical Committee of the Animal Research Service of the Military Hospital Gómez Ulla of Madrid and also by the Environment, Local Administration and Territorial Organization Office of Madrid Autonomy (order number PROEX 065/17). In this project, we used 24 male Wistar rats with a weight ranging from 200 to 250 g. Rats were housed in groups of two and given fourteen days to acclimate to the housing facility. Environmental conditions were a temperature of 20 ± 2 °C, humidity of 55 ± 10%. Animals were housed in cages and given access to rat maintenance food and water ad libitum. All sections of this report adhere to the ARRIVE (Animal Research: Reporting of in Vivo Experiments) guidelines for reporting animal research [18]. A completed ARRIVE guidelines checklist is included in Checklist S1.

For random allocation, we employed the EPIDAT 3.1 (Dirección Xéral de Saude Pública, Galicia, Spain) statistical package to generate a table of random numbers in three groups of eight rats and the control.

The determination of the sample size is based on similar research studies in which the tissue response to different biomaterials was studied, when these were implanted for short, medium and long periods [22,23,24].

### 2.2. Anaesthesia

The animals were anesthetized with a dissociative anesthetic, ketamine hydrochloride (Ketamidor^®^ 100 mg/mL, Karizoo S.A., Caldes de Montbui, Spain) at a dose of 75 mg/kg intramuscularly in combination with an alpha-2 agonist, medetomidine (Domtor^®^ 1 mg/mL, Orion Corporation, Espoo, Finland) at a dose of 0.5 mg/kg intramuscularly.

An opioid derivative, buprenorphine (Bupaq^®^ 0.3 mg/mL, (Karizoo S.A. Caldes de Montbui, Spain)), at a dose of 0.05 mg/kg administered subcutaneously, was used as intra- and postoperative analgesic medication. All animals, as a prophylactic measure against possible infections, were administered intramuscularly a broad-spectrum antibiotic with bactericidal action on Gram-negative and Gram-positive microorganisms, enrofloxacin (Syvaquinol^®^ 100 mg/mL (Laboratorios Syva, S.A.U. León., Spain)) at a dose of 5 mg/kg intramuscularly. Once anesthetized, they were placed in the prone position and immobilized on a cork board on a surgical table.

### 2.3. Surgical Protocol

The rats were shaved along the back and the lines where the incision would be made for the implantation of the biomaterials were marked equidistantly. Prior to the incision, the marked areas were cleaned and disinfected with povidone iodine (Betadine^®^ dermal solution 10 g/100 mL (Meda Pharma S.L., Madrid, Spain).

Four 10 mm incisions were made with a #15 scalpel blade (Surgical Disposable Scalpel # 15, Aesculap, Braun, Tuttlingen, Germany). Next, with blunt-tipped dissection scissors (Tekno 8365, Tekno Medical, Tuttlingen, Germany), a 20-mm deep pocket was created to obtain sufficient space in which to deposit the implant.

The implants consisted of 1.5 mm diameter sterile polyethylene tubing portions (BD ConnectaTM, Becton Dickinson Infusion Therapy AB). The polyethylene tubing was cut with a #15 scalpel blade (Surgical Disposable Scalpel # 15, Aesculap, Braun, Tuttlingen, Germany) into 5 mm long portions. No disinfection treatment was necessary for the tubes since they were sterile and packaged before use.

Four polyethylene tubes were implanted in each of the rats; three of them had been filled with the biomaterials under study, and the fourth was empty as a negative control of the histological reaction. The tubes were placed with a distance of at least 2 cm between them to avoid interference in the tissue response. A skin suture was made in each of the incisions with 2/0 braided silk (Silkam^®^, Braun, Tuttlingen, Germany). Four different groups were obtained: 1. Control. 2. MTA Angelus^®^ (Angelus, Londrina, Brazil). 3. MTA Angelus ^®^ G3. 4. Theracal LC^®^ (Bisco, Schaumburg, IL, USA).

### 2.4. Sacrifice and Histological Analysis

Once the period corresponding to each of the groups had ended, the animals were euthanized. To perform this, all the animals were administered a combination of a dissociative anesthetic, ketamine hydrochloride (Ketolar^®^ 100 mg/mL, (Pfizer Inc., New York, NY, USA)), at a dose of 75 mg/kg and an alpha-2 agonist, medetomidine (Domtor^®^ 1 mg/mL, Orion Corporation, Espoo, Finland) at doses of 0.5 mg/kg of weight intramuscularly (IM), prior to intracardiac (IC) administration of sodium pentobarbital (Dolethal^®^ 200 mg/mL, Vetoquinol SA, Lure, France). Three times the anesthetic dose is usually recommended. As confirmation and after the administration of drugs, all animals underwent a cervical dislocation. Verification of death was carried out by recognition of the animals [25].

To obtain the samples, the dorsal region of the rats was shaved in the groups in which the implantation of the biomaterials had been 30 and 45 days. In the 15-day group, it was not necessary. Subsequently, with a number 15 scalpel blade (Surgical Disposable Scalpel nº 15, Aesculap, Braun, Tuttlingen, Germany) and sterile scissors (Tekno 8365, Tekno Medical, Tuttlingen, Germany), the skin and subcutaneous connective tissue around the implant, including it, were resected. The surface area of the samples was approximately 2 cm × 2 cm. The samples were then placed in a 10% buffered formaldehyde solution (Histogen 40, Serosep Ltd., Limerick, Ireland) until they were processed in the pathology laboratory.

**Figure 1:**
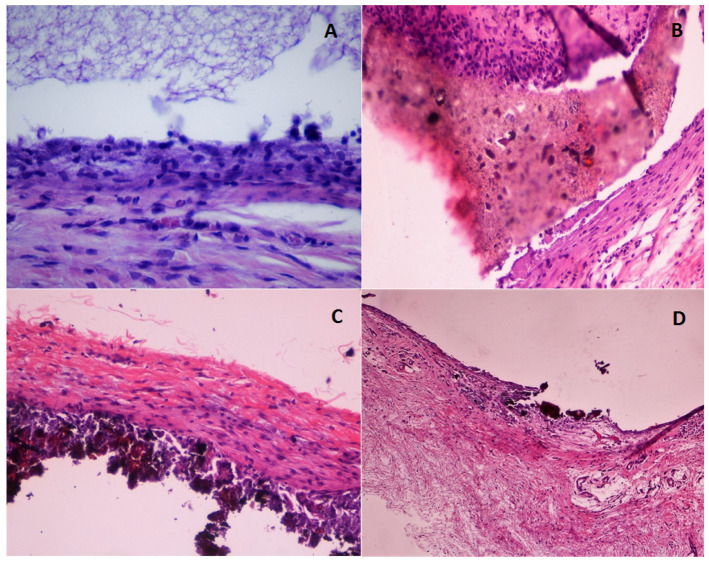
Hematoxylin–eosin stain. (**A**): Control. Fibrous capsule 30 days (×400). (**B**): Theracal LC fibrous capsule 45 days (×200). (**C**): MTA fibrous capsule surface calcification 45 days (×200). (**D**): MTA-G3 fibrous capsule calcification 45 days (×100).

The evaluation of the histological preparations was carried out by two observers, by double blindness. In cases where there was a discrepancy, a third observer was consulted (Figure 1).

To carry out the evaluation, the criteria of Taha et al. was used [26]. They are based on the criteria established by Parirokh et al. [27]. The criteria used by Taha et al. were slightly modified in our evaluation (Table 1).

### 2.5. Statistical Analysis

All the results were analyzed with the SPSS^®^ 20.0 (IBM, Armonk, NY, USA) for Windows statistical package. First, the results were described with percentages and then the potential association between the agent and the histological data were evaluated with the Mann–Whitney U test for dichotomic and quantitative variables and the Kruskal–Wallis test for politomic and quantitative variables.

## 3. Results

**Figure 2:**
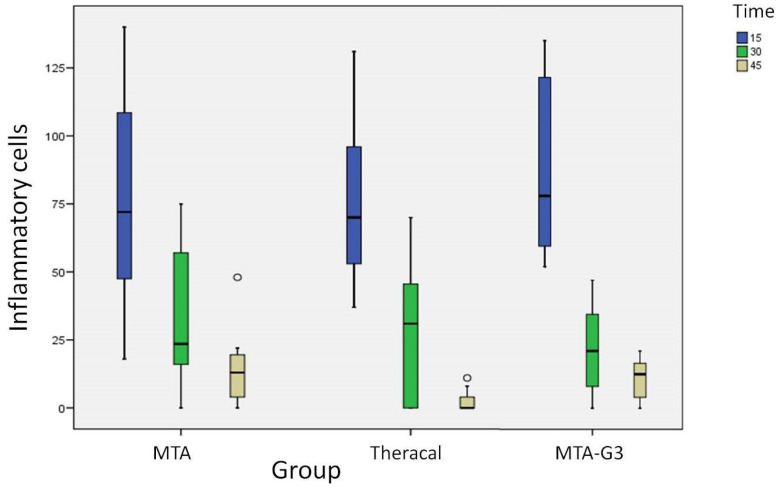
Number of inflammatory cells MTA, Theracal and MTA-G3. Differences between periods. The density decreases with the duration of the implant.

The intensity of the inflammatory reaction was evaluated with the number of inflammatory cells for the microscopic field of observation at 400× magnification. The density decreases with the duration of the implant. This decrease is statistically significant in tube implants with MTA (*p* = 0.004), Theracal LC (*p* = 0.001) and it is also significant with MTA-G3 (*p* ≤ 0.001) (Figure 2 and Table 2).

**Figure 3:**
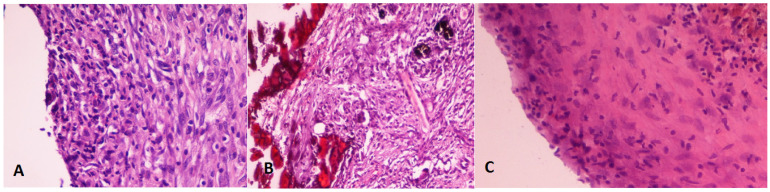
Type of cells predominant inflammatory response: lymphocytes, macrophages, and/or polymorphonuclear cells. Hematoxylin–eosin stain. (**A**): Theracal 15 days (40×). (**B**): MTA-G3 15 days (10×). (**C**): MTA 15 days (20×).

If we compare the inflammatory reaction to the three materials according to periods, in the samples of 15 and 30 days, no differences were found between the three materials. However, in the 45-day samples, the differences are significant (*p* = 0.021). In this period, the median value of inflammatory cells with MTA turned out to be 13 cells, Md (IQR) = 13 (18.75), in Theracal LC it was 0 cells, Md (IQR) = 0 (6) and in the samples with MTA-G3 it was 12.5 cells (15.25) (Table 3 and Figure 3).

Regarding qualitative variables studied to evaluate the tissue response, we observed that in the 15-day period, no significant differences were found in any of the parameters studied. The foreign body reaction and the appearance of calcifications had an identical frequency distribution (Table 4).

In the 30-day period, significant differences were found in the fibroblast reaction and in the appearance of dystrophic calcifications between MTA, MTA-G3 and Theracal (Table 5).

In the 45-day period, we found that 75% of the samples from the Theracal LC implantation did not show signs of inflammation. In contrast, in the MTA and MTA-G3 samples, there was no inflammation in only two samples (25%). However, the difference can be explained by chance (*p* = 0.132) (Table 6).

**Figure 4:**
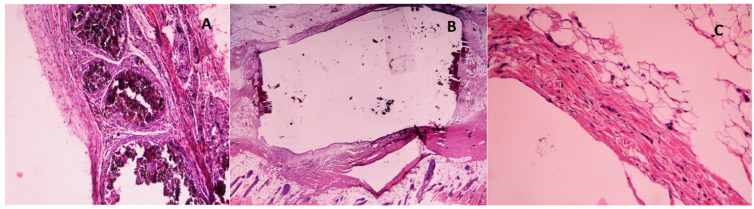
Hematoxylin–eosin stain. (**A**): Dystrophic calcification in the area of contact with MTA 30 days (200×). (**B**): Calcification on the open ends of the MTA-G3 polyethylene tube 30 days (40×). (**C**): 136 µm thick fibrous capsule Theracal 45 days (200×).

On the other hand, a comparative study of the formation of calcifications was carried out, without taking into account the implantation period. Dystrophic calcification occurred in 75% of the samples with MTA, 37.5% in those with Theracal LC implantation and 45.8% in the MTA-G3 samples, finding differences between MTA and MTA-G3/Theracal (Table 7 and Figure 4).

**Figure 5:**
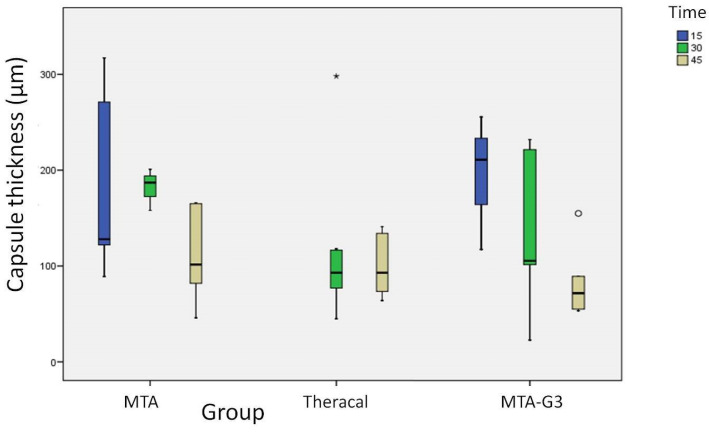
MTA, Theracal and MTA-G3 fibrous capsule thickness. Differences between periods.

Regarding the thickness of the fibrous capsule, no statistically significant differences were found in MTA of 15, 30 and 45 days (*p* = 0.08), nor in MTA-G3 (*p* = 0.068). With respect to Theracal LC, none of the 15-day histological sections presented mature capsules, and no significant differences were found in the thickness of the capsules in the 30-day samples compared to the 45-day samples (*p* = 0.834) (Figure 5 and Table 8). If we compare the median value of the measurement in microns of the three materials with each other, by periods, we obtain that there is a significant difference between MTA/MTA-G3 and Theracal LC in the 30-day samples (*p* = 0.041). The capsule formed against MTA and MTA-G3 was found to be 94 µm thicker (Table 9).

## 4. Discussion

The tissue response with MTA was characterized by a chronic, moderate inflammation, which with the passage of time lost intensity until it practically disappeared after 45 days of contact with the material. The initial degree of inflammation can be a response to several factors such as high pH, the temperature generated during setting, and the generation of cytokines, such as interleukin-1 and interleukin-6 [28]. The connective tissue was arranged in the form of collagen fibbers that formed fibrous capsules around the tube and its open ends that had direct contact with the material. The most significant histological changes were characterized by the formation of dystrophic calcifications. These were already evident in 62.5% of the samples on day 15, appearing in all the samples in the longest periods. Bramante et al. [23] differ from our results in that at 15 days, they only observed a few inflammatory cells and it made no reference to calcifications in any of the groups. Holland et al. [7] describe the existence of birefringent formations in polarized light, possibly calcite crystals. These formations are consistent with the calcifications in the tissue found in our study. Conversely, the proportion of samples with dystrophic calcifications in our study is much higher than that of Taha et al. [26]; this could be due to the use of a different variety of MTA (Angelus vs. ProRoot MTA). Angelus MTA composition is 5% more Portland cement than ProRoot MTA. In addition, the shape and size of the particles is less homogeneous [29]. Shahi et al. [5] describe the presence of a chronic inflammatory infiltrate, collagen fibbers and fibrosis at 21 days. Parirokh et al. [30] describe calcium precipitations in 33% of the samples with GMTA and 22% of the samples with WMTA. In our study, we also found the presence of calcifications in much higher percentages. Calcifications were seen in 75% of the samples with MTA and in 54% of the samples with MTA-G3.

Martínez Lalis et al. [22] report a chronic inflammatory infiltrate and the formation of organized fibrous tissue whose results do not contradict ours. Yaltirik et al. [6] agree with our results, since in the 15-day samples they describe the presence of dystrophic calcifications and a moderate infiltration of inflammatory cells, in which macrophages and multinucleated giant cells can be seen; at 30 days, they observe calcifications and the formation of fine fibrous capsules, together with a decrease in inflammatory cells.

The formation of calcium structures in the subcutaneous connective tissue is a sign of osteoinductive activity of the evaluated material [31], in this case the MTA. Calcite crystals are formed due to the reaction of calcium from calcium hydroxide with carbon dioxide from connective tissue. Calcium hydroxide originates from calcium phosphate and calcium oxide, which releases MTA [5].

The biocompatibility of MTA is also related to its property to release calcium ions, since it causes an elevation in the pH of the medium and does not allow bacterial growth [32]. 

We have not found any work in which the implantation in the subcutaneous connective tissue of MTA was carried out with the G3 glass. López-Píriz et al. coated with G3 implants used in Beagle dogs, demonstrating the biocidal nature of glass, and its ability to prevent bone resorption [20], but its effectiveness in reducing response in tissue has not been demonstrated.

The tissue response to MTA-G3 did not differ from MTA in any of the parameters studied and the addition of glass did not improve, nor did it worsen the tissue response to MTA. Differences were only found in the 30-day group, where MTA presented more calcifications than MTA-G3. It is possible that 25% less cement than the mix has in its composition is the main cause. Surely if the comparison between MTA and G3 were made at 100%, the difference would be much more important.

The use of Theracal did not elicit any macroscopically visible lesions. At 15 days, the samples presented a chronic inflammation of moderate or severe intensity, and at 45 days it was scarce and practically disappeared in almost all the samples. In the statistical study, no statistically significant differences were found.

The difference between median values was only 13 inflammatory cells. Considering that no significant differences were found between type and intensity of inflammation between MTA and negative controls, we have to subtract significance from this small difference between MTA and Theracal LC.

Some authors conducted in vitro cytotoxicity studies and described greater cell viability in experiments with MTA than in those using Theracal LC; we believe that it must be considered that in the living model, the cellular response, inflammatory phenomena and reparative actions may be determining factors of the final result and the conclusions of the study [33].

Regarding the fibroblastic reaction, the connective tissue was organized into collagen fibbers and fibroblasts that were forming palisades. At 15 days, none of the fibrous capsules were still mature, but at 30 days, all of the samples had a capsule formed around the Theracal LC and around the polyethylene tubes. The thickness of the capsules that were formed against Theracal LC was 94 µm lower (difference between medians) than the capsules formed with MTA. At 45 days, the thickness of the capsules formed against Theracal was practically the same. Martínez-Lalis et al. also measured the thickness of the fibrous capsule formed around the implanted material [22]. The formation of fibrous connective tissue around the implanted material indicates that it has been well tolerated by the surrounding tissues [6]. Parirokh et al. stated that the formation of a fibrous capsule and the progressive decrease in its thickness over time is one of the signs of biocompatibility [30].

Another of the findings found in the Theracal samples were calcifications. Observing that the proportion of samples with calcifications was higher at 15 days (62.5%) than at 45 days (25%), the samples were compared regardless of the period. The samples with MTA^®^ and MTA/G3 presented two times more dystrophic calcifications than with Theracal LC^®^. If we take into account that MTA^®^ has 35% more Portland cement in its composition than Theracal LC^®^, the content of calcium salts is much higher in MTA. The difference in calcium ions in the composition of both materials could explain the greater formation of calcifications in the samples with MTA. Torneck et al. stated that calcium ions would activate ATP, which would play an important role in the mineralization process in tissues [34].

Gandolfi et al. observed, however, that Theracal LC^®^ released more calcium ions during the first few days than ProRoot MTA^®^ in an experimental in vitro study [11], demonstrating that Theracal LC^®^ was capable of releasing calcium and hydroxyl ions for at least 28 days.

One of the possible advantages of using Theracal as a potential root-end filling would be the combination with moisture necessary for setting, since in these procedures it is very complex to achieve a lack of total humidity and, in addition, polymerization would allow us to check the plug in situ. The fact that it is light-cured is not a problem during root-end surgery, as has been demonstrated in the literature [35]. 

Hinata et al. evaluated the abilities of TheraCal LC to produce apatite-like precipitates after being subcutaneously implanted into rats at 7, 14 and 28 days post-implantation through scanning electron microscopy (SEM-EPMA). This study concluded that the thickness of the Ca-and P-rich region was thicker in MTA versus Theracal [36]. The deposition of mineralized materials (dystrophic mineralization) adjacent to materials has been considered to be a crucial event during mineralized tissue repair [37]; the present study has analyzed samples in 15, 30 and 45 days and the presence of Ca and P ions have not been evaluated, but calcifications were similar finding the presence of a greater number of calcifications in MTA versus Theracal. Fibrous capsule thickness increased over time, which could confirm that the release of Theracal is sustained over time.

One of the limitations of the study is the subjective nature of the parameters evaluated in the observation of the histological preparations. Although the established criteria are objective, the data recorded by pathologists may vary depending on the microscopic field observed or the histological section studied. Furthermore, the results cannot be extrapolated to man, based solely on the data obtained in the experimental animals, but the implantation of the experimental materials in the subcutaneous connective tissue of the rat is considered by many authors as a valid procedure for the study of its biological properties [38,39].

## 5. Conclusions

Theracal LC as well as the MTA Angelus were well tolerated when implanted in the rat’s subcutaneous connective tissue.

Theracal LC and MTA with the bioactive glass G3, caused an inflammatory reaction, which at 15 days was chronic and decreased in intensity, until almost disappearing after 45 days of contact. No edema or necrosis were observed. A fibrous capsule was formed, which once mature, was decreasing in thickness, around the polyethylene tubes and the biomaterials evaluated.

Angelus MTA induces the formation of dystrophic calcifications twice as much as Theracal LC. The addition of bioglass G3 in 25% did not affect the greater or lesser occurrence of calcifications. No problem was found histologically for the direct contact use of Theracal LC, and the mixing of MTA with 25% bioactive G3 glass, with the connective tissue.

## Figures and Tables

**Table 1:** Criteria used to carry out the evaluation. They are based in Taha et al. but they were slightly modified in our evaluation.

		Grade					
Appearance	Category	Value 0	Value 1	Value 2	Value 3	Value 4	Value 5
Inflammation	Kind	Absence of both types (Acute and chronic)	Chronicle	Sharp	Both types		
	Intensity (concentration of inflammatory cells per field ×40)	Inflammatory cells are not detected	Less than 25 cells (rare)	Between 25 and 50 cells (mild)	Between 50 and 75 cells (moderate)	More than 75 cells (severe)	
	Extension	Inflammatory cells are not detected	Inflammatory cells are located in the superficial layer of the capsule	Inflammatory cells located within the limits of the capsule	Inflammatory cells beyond the capsule	There is no encapsulation. Cells are limited to the contact area	There is no encapsulation. Inflammatory cells spread outside the contact area
Fibroblastic reaction	Connective tissue capsule	Absent	Immature form	Mature, thin form (<150 µm)	Mature, coarse form (≥150 µm)		
Foreign body reaction	Giant cells or foreign body eosinophils	Absence	Presence				
Capillary reaction	Congestion in blood vessels	Absence	Presence				
	Tissue edema	Absence	Limited areas of tissue edema	Widespread areas of tissue edema			
Calcification		Absence	Presence				
Necrosis		Absence	Presence				

**Table 2:** Number of inflammatory cells MTA, Theracal and MTA-G3. Differences between periods.

PARAMETER	MATERIAL	DAY 15	DAY 30	DAY 45	*p*
Number of inflammatory cells Md(RIQ)	MTA	72 (74.5)	23.5 (47.5)	13 (18.75)	0.004 *
	MTA-G3	78 (70.5)	21 (35.75)	12.5 (15.25)	<0.001 *
	THER.	70 (58)	31 (46.75)	0 (6)	0.001 *

*, Kruskal–Wallis.

**Table 3:** Number of inflammatory cells MTA, Theracal and MTA-G3. Differences between materials according to periods.

PARAMETER	DAYS	MTA	THER.	MTA-G3	*p*
Number of inflammatory cells Md(RIQ)	15	72 (74.5)	70 (58)	78 (70.5)	0.916 *
	30	23, 5 (47.5)	31 (46.75)	21 (35.75)	0.711 *
	45	13 (18.75)	0 (6)	12.5 (15.25)	0.021 *

*, Kruskal-Wallis.

**Table 4:** Qualitative variables MTA, MTA-G3 and Theracal, 15 days.

PARAMETER	GRADE	MTA	THER.	MTA-G3	Control	*p*
Type of inflammation *n*(%)	Absence	0 (0)	0 (0)	0 (0)	0 (0)	-
	Chronic	8 (100)	8 (100)	8 (100)	8 (100)	
Intensity *n*(%)	Limited	1 (12.5)	0 (0)	0 (0)	0 (0)	
	Mild	1 (12.5)	2 (25)	0 (0)	0 (0)	0.551 *
	Moderate	3 (37.5)	4 (50)	4 (50)	5 (62.5)	
	Severe	3 (37.5)	2 (25)	4 (50)	3 (37.5)	
Number of inflammatory cells Md(RIQ)		72 (74.5)	70 (58)	78 (70.50)	74.5 (58.75)	0.938 **
Extension *n*(%)	Not detected	0 (0)	0 (0)	0 (0)	0 (0)	
	On capsule surface	0 (0)	0 (0)	0 (0)	0 (0)	
	Inside the capsule	5 (62.5)	2 (25)	0 (0)	3 (37.5)	
	Beyond the capsule	3 (37.5)	3 (37.5)	6 (75)	5 (62.5)	0.058 *
	In contact area	0 (0)	3 (37.5)	2 (25)	0 (0)	
	Extended	0 (0)	0 (0)	0 (0)	0 (0)	
Capsule *n*(%)	Absence	0 (0)	1 (12.5)	2 (25)	0 (0)	
	Immature	3 (37.5)	7 (87.5)	3 (37.5)	5 (62.5)	0.123 *
	Fine	3 (37.5)	0 (0)	1 (12.5)	3 (37.5)	
	Thick	2 (25)	0 (0)	2(25)	0 (0)	
Capsule thickness Md(RIQ)		128 (188.5)	0 (0)	241 (58)	83 (87)	0.212 **
Strange body *n*(%)	Yes	4 (50)	4 (50)	5 (62.5)	2 (25)	0.497 *
	No	4 (50)	4 (50)	3 (37.5)	6 (75)	
Calcification *n*(%)	Yes	5 (62.5)	5 (62.5)	7 (87.5)	0 (0)	0.004 *
	No	3 (37.5)	3 (37.5)	1 (12.5)	8 (100)	
Congestion *n*(%)	Yes	1 (12.5)	2 (25)	1 (12.5)	2 (25)	0.845 *
	No	7 (87.5)	6 (75)	7 (87.5)	6 (75)	
Edema *n*(%)	Yes	0 (0)	0 (0)	0 (0)	0 (0)	-
	No	8 (100)	8 (100)	8 (100)	8 (100)	
Necrosis *n*(%)	Yes	0 (0)	0 (0)	0 (0)	0 (0)	-
	No	8 (100)	8 (100)	8 (100)	8 (100)	

*, χ^2^ Pearson, **, Kruskal-Wallis.

**Table 5:** Qualitative variables MTA, Theracal and MTA-G3, 30 days.

PARAMETER	GRADE	MTA	THER.	MTA-G3	Control	*p*
Type of inflammation *n*(%)	Absence	1 (12.5)	3 (37.5)	2 (25)	2 (25)	0.612
	Chronic	7 (87.5)	5 (62.5)	6 (75)	6 (75)	
Intensity *n*(%)	Limited	1 (12.5)	3 (37.5)	2 (25)	2 (25)	
	Mild	4 (50)	1 (12.5)	4 (50)	4 (50)	0.532 *
	Moderate	1 (12.5)	3 (37.5)	2 (25)	2 (25)	
	Severe	2 (25)	1 (12.5)	0 (0)	0 (0)	
Number of inflammatory cells Md(RIQ)		23.5 (47.5)	31 (46.75)	21 (35.75)	38.50 (58)	0.620 **
Extension *n*(%)	Not detected	1 (12.5)	3 (37.5)	2 (25)	2 (25)	
	On capsule surface	0 (0)	0 (0)	0 (0)	0 (0)	
	Inside the capsule	5 (62.5)	1 (12.5)	6 (75)	3 (37.5)	
	Beyond the capsule	1 (12.5)	4 (50)	0 (0)	3 (37.5)	0.166 *
	In contact area	1 (12.5)	0 (0)	0 (0)	0 (0)	
	Extended	0 (0)	0 (0)	0 (0)	0 (0)	
Capsule *n*(%)	Absence	2 (25)	0 (0)	2 (25)	0 (0)	
	Immature	2 (25)	0 (0)	0 (0)	0 (0)	0.016 *
	Fine	0 (0)	7 (87.5)	4 (50)	7 (87.5)	
	Thick	4 (50)	1 (12.5)	2 (25)	1 (12.5)	
Capsule thickness Md(RIQ)		187 (32.25)	93 (40.75)	120.5 (162.5)	68 (81.5)	0.047 **
Strange body *n*(%)	Yes	0 (0)	1 (12.5)	1 (12.5)	1 (12.5)	0.545 *
	No	8 (100)	7 (87.5)	7 (87.5)	7 (87.5)	
Calcification *n*(%)	Yes	8 (100)	2 (37.5)	2 (37.5)	0 (0)	<0.001 *
	No	0 (0)	6 (75)	6 (75)	8 (100)	
Congestion *n*(%)	Yes	0 (0)	0 (0)	0 (0)	0 (0)	-
	No	8 (100)	8 (100)	8 (100)	8 (100)	
Edema *n*(%)	Yes	0 (0)	0 (0)	0 (0)	0 (0)	-
	No	8 (100)	8 (100)	8 (100)	8 (100)	
Necrosis *n*(%)	Yes	0 (0)	0 (0)	0 (0)	0 (0)	-
	No	8 (100)	8 (100)	8 (100)	8 (100)	

*, χ^2^ Pearson, **, Kruskal-Wallis.

**Table 6:** Qualitative variables MTA, Theracal and MTA-G3, 45 days.

PARAMETER	GRADE	MTA	THER.	MTA-G3	Control	*p*
Type of inflammation	Absence	2 (25)	6 (75)	2 (25)	3 (37.5)	0.188 *
*n*(%)	Chronic	6 (75)	2 (25)	6 (75)	5 (62.5)	
	Absence cells	2 (25)	6 (75)	2 (12.5)	3 (37.5)	
Intensity	Limited	5 (62.5)	2 (25)	6 (75)	4 (50)	
*n*(%)	Mild	1 (12.5)	0 (0)	0 (0)	0 (0)	0.251 *
	Moderate	0 (0)	0 (0)	0 (0)	1 (12.5)	
	Severe	0 (0)	0 (0)	0 (0)	0 (0)	
Number of inflammatory cells Md(RIQ)		13 (18.75)	0 (6)	12.5 (15.25)	12 (19.5)	0.081 **
Extension *n*(%)	Not detected	2 (25)	6 (75)	1 (12.5)	3 (37.5)	
	On capsule surface	0 (0)	0 (0)	1 (12.5)	0 (0)	
	Inside the capsule	4 (50)	2 (25)	1 (12.5)	4 (50)	
	Beyond the capsule	1 (12.5)	0 (0)	3 (37.5)	0 (0)	0.125 *
	In contact area	1 (12.5)	0 (0)	1 (12.5)	1 (12.5)	
	Extended	0 (0)	0 (0)	0 (0)	0 (0)	
Capsule *n*(%)	Absence	2 (25)	0 (0)		1 (12.5)	
	Immature	0 (0)	0 (0)		0 (0)	0.274 *
	Fine	4 (50)	8 (100)	2 (25)	7 (87.5)	
	Thick	2 (25)	0 (0)	0 (0)	0 (0)	
Capsule thickness Md(RIQ)		101.5 (92.25)	93 (65.25)	82 (58.25)	43 (18)	0.003 **
Strange body	Yes	0 (0)	0 (0)	0 (0)	1 (12.5)	0.396 *
*n*(%)	No	8 (100)	8 (100)	8 (100)	7 (87.5)	
Calcification	Yes	5 (62.5)	2 (37.5)	2 (37.5)	0 (0)	0.048 *
*n*(%)	No	3 (37.5)	6 (75)	6 (75)	8 (100)	
Congestion	Yes	0 (0)	0 (0)	0 (0)	0 (0)	-
*n*(%)	No	8 (100)	8 (100)	8 (100)	8 (100)	
Edema	Yes	0 (0)	0 (0)	0 (0)	0 (0)	-
*n*(%)	No	8 (100)	8 (100)	8 (100)	8 (100)	
Necrosis	Yes	0 (0)	0 (0)	0 (0)	0 (0)	-
*n*(%)	No	8 (100)	8 (100)	8 (100)	8 (100)	

*, χ^2^ Pearson, **, Kruskal-Wallis.

**Table 7:** Presence of MTA, Theracal and MTA-G3 calcifications.

PARAMETER	GRADE	MTA	THER.	MTA-G3
Calcification	Yes	18 (75)	9 (37.5)	11 (45.8)
*n* (%)	No	6 (25)	15 (62.5)	13 (54.2)

**Table 8:** MTA, Theracal and MTA-G3 fibrous capsule thickness. Differences between periods.

PARAMETER	MATERIAL	DAY 15	DAY 30	DAY 45	*p*
Thickness (µm) Md(RIQ)	MTA	128 (188.5)	187 (32.25)	101.5 (92.25)	0.08 *
	THER.		93 (40.75)	93 (65.25)	0.834 **
	MTA-G3	241 (158)	120.5 (162.5)	82 (58.25)	0.068 *

*χ^2^ Pearson, **, Kruskal-Wallis.

**Table 9:** MTA, Theracal and MTA-G3 fibrous capsule thickness. Differences between materials according to period.

PARAMETER	DAYS	MTA	THER.	MTA-G3
Thickness (µm) Md(RIQ)	15	128 (188.5)		241 (158)
	30	187 (32.25)	93 (40.75)	120.5 (162.5)
	45	101.5 (92.25)	93 (65.25)	82 (58.25)

## Data Availability

The datasets used and/or analyzed during the current study are available from the corresponding author on reasonable request.

## Supplementary Materials

The following are available online at https://www.mdpi.com/article/10.3390/jcm10122536/s1, Checklist S1: ARRIVE guidelines checklist.
